# The Roles of Ubiquitin in Mediating Autophagy

**DOI:** 10.3390/cells9092025

**Published:** 2020-09-02

**Authors:** Zhangyuan Yin, Hana Popelka, Yuchen Lei, Ying Yang, Daniel J. Klionsky

**Affiliations:** 1 Life Sciences Institute, University of Michigan, Ann Arbor, MI 48109, USA; zyyin@umich.edu (Z.Y.); popelka@umich.edu (H.P.); yclei@umich.edu (Y.L.); yingyan@umich.edu (Y.Y.); 2 Department of Molecular, Cellular and Developmental Biology, University of Michigan, Ann Arbor, MI 48109, USA

**Keywords:** autophagy, lysosome, selective autophagy, ubiquitin, ubiquitination

## Abstract

Ubiquitination, the post-translational modification essential for various intracellular processes, is implicated in multiple aspects of autophagy, the major lysosome/vacuole-dependent degradation pathway. The autophagy machinery adopted the structural architecture of ubiquitin and employs two ubiquitin-like protein conjugation systems for autophagosome biogenesis. Ubiquitin chains that are attached as labels to protein aggregates or subcellular organelles confer selectivity, allowing autophagy receptors to simultaneously bind ubiquitinated cargos and autophagy-specific ubiquitin-like modifiers (Atg8-family proteins). Moreover, there is tremendous crosstalk between autophagy and the ubiquitin-proteasome system. Ubiquitination of autophagy-related proteins or regulatory components plays significant roles in the precise control of the autophagy pathway. In this review, we summarize and discuss the molecular mechanisms and functions of ubiquitin and ubiquitination, in the process and regulation of autophagy.

## 1. Introduction

The proper balance between synthesis and degradation that maintains cellular homeostasis is essential for all eukaryotic cells. Proteins, metabolites and organelles are continuously generated and degraded in a delicate equilibrium to support cellular growth, function, development and survival. Disruption of the degradation/recycling process could cause the accumulation of damaged, or superfluous proteins and organelles, which can in turn perturb related cellular processes, harm the cells and even induce dysfunction at the organ level. Therefore, it is of great importance to understand the mechanisms and regulation of the two major intracellular degradation pathways: autophagy and the ubiquitin-proteasome system (UPS).

Autophagy is a dynamic recycling process that involves degradation of cytoplasmic components in the vacuole/lysosome. There are three primary types of autophagy: microautophagy, chaperone-mediated autophagy and macroautophagy, which mainly differ in the types of cargo they degrade and how these cargos are delivered [1]. This review focuses on macroautophagy (hereafter referred to as autophagy), which is characterized by the de novo formation of a transient double-membraned compartment, termed a phagophore, that sequesters intracellular cargo including cytosol, protein aggregates and organelles. The phagophore matures into a closed autophagosome that subsequently fuses with the vacuole/lysosome, allowing degradation of the enclosed cargo, and release of the breakdown products for reuse by the cell. Genetic screens in yeast identified a set of autophagy-related (*ATG*) genes and pioneered the understanding of the autophagy machinery [2]; not surprisingly, the genes encoding the core machinery are highly conserved among more complex eukaryotes. Through reverse genetic approaches in various cellular and animal models, more physiological and pathophysiological roles of autophagy have been uncovered [3]. Although autophagy primarily acts as an inducible rapid adaptation mechanism to cope with environmental adversity, such as starvation or hypoxia, the constitutive turnover of cytoplasmic contents through basal autophagy in nutrient-rich conditions is also crucial for cellular homeostasis.

Because any portion of the cytoplasm can be randomly engulfed by phagophores, autophagy has long been considered nonselective. However, many studies have revealed that aggregates, organelles and invading pathogens can be targeted in a highly selective manner. Selective autophagy occurs constitutively but can also be induced in response to damaged or dysfunctional organelles and changing nutrient conditions. The selectivity of cargo recognition relies on the unique cargo receptors, which tether cargos to the phagophore by the simultaneous binding of cargo and ubiquitin-like (UBL) modifiers, comprised of the Atg8-family proteins [4]. Of note, ubiquitination of cargos/substrates is often employed as a label to confer selectivity through the interaction of this moiety with receptors.

Ubiquitination is the process of attaching ubiquitin, a highly conserved 76 amino acid globular protein, to protein substrates via covalent conjugation [5]. This modification involves a multistep reaction that requires the sequential action of three types of enzymes: E1 (ubiquitin-activating enzyme), E2 (ubiquitin-conjugating enzyme) and E3 (ubiquitin ligase). The E1 activates ubiquitin and forms a ubiquitin adenylate. Next, activated ubiquitin is transferred to a cysteinyl residue in the E2. Finally, the E3 transfers ubiquitin to the Lys residues on substrates, although sometimes Cys, Ser and Thr residues are the sites of attachment [6,7,8]. For many organisms, there is only one E1, but a variety of E2 and especially E3 enzymes [5]. For example, in *S. cerevisiae*, there are approximately 80 genes found or predicted to encode E3 ligases; in humans, this number was estimated to be approximately 600–700 [9]. Consequently, substrate recognition and much of the selectivity are dependent on E3. Ubiquitin that has already been conjugated to a substrate can be further modified by post-translational modifications, such as additional ubiquitination, phosphorylation, acetylation and SUMOylation (covalent attachment of small UBL SUMO proteins), resulting in increased complexity of the ubiquitin signaling [10].

Ubiquitin signals can be recognized, processed and bound by specialized ubiquitin-binding domains (UBDs), which will direct the substrates to downstream processes [11]. For example, the most common fate of poly-ubiquitinated proteins is to be recognized and delivered by receptors to the 26S proteasome for degradation, which enables the rapid selective recycling of thousands of different proteins, and thus in turn affects many aspects of cellular physiology. Specific interaction between ubiquitinated substrates and selective autophagy receptors facilitates autophagic degradation. Finally, ubiquitin–UBD interactions can also modulate non-degradative processes, including DNA repair, regulation of protein activity/location and immune-signaling pathways [12]. Ubiquitination is a dynamic and reversible process; the attached ubiquitin can be removed by de-ubiquitinating enzymes, thus allowing for spatiotemporal regulation of proteolysis and cell signaling [13].

In this review, we describe the two ubiquitin-like conjugation systems in autophagy and further discuss the potential ubiquitin-like folds in Atg5. We also consider the molecular and functional crosstalk between autophagy and the UPS. Last, we review and discuss available evidence for the roles of ubiquitination in autophagy, in particular, ubiquitin-dependent signals in selective autophagy, and how ubiquitination and de-ubiquitination regulate the levels of Atg proteins and autophagy regulators.

## 2. Ubiquitin-like Conjugation Systems in Autophagy

**Figure 1:**
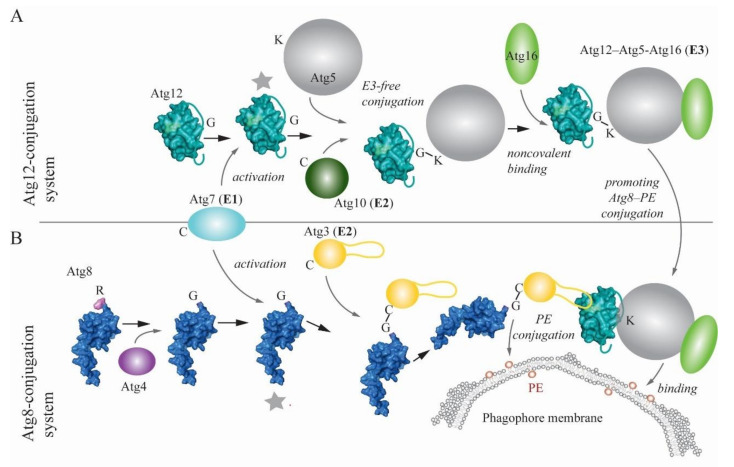
Schematic representation of the Atg8- and Atg12-conjugation systems in yeast. (**A**) Atg12 is activated by Atg7, the E1-like (activating) enzyme, and then transferred to Atg10, the E2-like (conjugating) enzyme. The Atg12–Atg10 intermediate interacts with Atg5, where the conserved lysine residue is covalently conjugated to the Atg12 C terminus in the E3-free (ligase) reaction. The Atg12–Atg5 binds noncovalently to Atg16. The resulting Atg12–Atg5-Atg16 complex acts as the E3-like enzyme in the Atg8–PE conjugation reaction. (**B**) Atg8 enters the conjugation reaction unprimed, due to the presence of a C-terminal arginine. Atg4 primes Atg8 by removing this last residue, leaving a C-terminal glycine exposed. As in the case of Atg12, Atg7 activates the Atg8 ubiquitin-like (UBL) domain by C-terminal adenylation, and then transfers Atg8 to the catalytic cysteine in the active site of Atg3. The Atg12–Atg5-Atg16 complex (E3-like) interacts with the Atg8~Atg3 intermediate, where a long flexible loop of Atg3 binds to a hydrophobic cavity on the surface of Atg12. This interaction enhances ligation of the C-terminal Gly in Atg8 to PE on the phagophore membrane. Atg8 and Atg12 are visualized using the crystal structures PDB ID: 2KQ7 and PDB ID: 3W1S, respectively. Gray stars denote activated states of molecules.

The autophagy pathway adopted the structural architecture of ubiquitin in a few proteins of the core machinery (i.e., those proteins that are required for autophagosome formation). Two of these ubiquitin-like proteins, Atg8 and Atg12 in yeast and MAP1LC3 (microtubule-associated protein 1 light chain 3—abbreviated LC3 hereafter)/GABARAP (GABA type A receptor-associated protein) and ATG12 in more complex eukaryotes, are substrates of two parallel, highly conserved ubiquitin-like conjugation reactions that are mediated by E1-E2-E3-like enzymes (Figure 1) [14,15,16,17,18]. Before Atg8/LC3/GABARAP enters this enzymatic cascade, the protein is primed by Atg4 (yeast)/ATG4 (more complex eukaryotes), which removes a C-terminal extension to expose a glycine residue. Subsequently, Atg7/ATG7 acts as an E1-like enzyme activating the inert C terminus of both UBL proteins, which enables covalent linkage of their C terminus to the catalytic cysteine of Atg7/ATG7. Afterwards, the E1-like protein delivers UBLs to the active site of E2-like (conjugating) enzymes, which differ depending on the ubiquitin-like conjugation system. The Atg12/ATG12 conjugation system utilizes Atg10/ATG10, whereas the conjugation system of Atg8-family proteins relies on Atg3/ATG3. The E2 Atg10/ATG10 directly interacts with Atg5/ATG5, allowing downstream ligation of a C-terminal glycine residue on Atg12/ATG12 to a single conserved lysine on Atg5/ATG5 in an E3-independent manner. The resulting Atg12–Atg5/ATG12–ATG5 conjugate binds noncovalently to Atg16/ATG16L1 and together, the complex acts as an E3-like (ligase) enzyme in the Atg8-family protein conjugation reaction [19]. The E3-like Atg12–Atg5-Atg16 in yeast, and analogously ATG12–ATG5-ATG16L1 in more complex eukaryotes, promotes transfer of Atg8-family proteins from the E2-like enzyme to the primary amino group of phosphatidylethanolamine (PE) on the phagophore membrane [17,20]. Structures and molecular mechanisms of interactions within the Atg7-Atg8-Atg3 and Atg7-Atg12-Atg10 complexes were revealed in detail by several studies [21,22,23,24,25,26], and are comprehensively summarized in a structure-focused review [17].

A very recent study elaborated on one of these mechanisms, specifically, on structural flexibility of Atg3 homologs [27]. Zheng et al. showed that a peptide within a long intrinsically disordered loop of Atg3 functions as an allosteric switch that restrains the catalytic loop of the molecule until binding of E1- or E3-like enzymes to this peptide releases, via intramolecular interactions, the inactive conformation [27]. Progress has also been made using biophysical and biochemical approaches that revealed a new molecular mechanism promoting lipidation of Atg8 homologs. These studies showed that human ATG3 and ATG16L1 utilize an amphipathic helix at the N terminus for insertion into the lipid bilayer [28,29]. New insights into the E3-like complex recently unmasked its novel functions, showing a more significant role than was previously ascribed to this complex. One study revealed that the E3-like complex binds autophagy cargo receptors. Specifically, Atg19 or Atg34 in yeast, and SQSTM1/p62 (sequestosome 1), CALCOCO2/NDP52 (calcium binding and coiled-coil domain 2) or OPTN (optineurin) in human cells utilize the Atg8-interacting motif (AIM)/LC3-interacting region (LIR) motif to interact with Atg5/ATG5. In vitro assessments with Atg19 showed that the Atg5-Atg19 AIM interaction competes with the Atg8-Atg19 AIM interaction [30]. Another study proposed that the Atg12–Atg5-Atg16 complex from yeast is involved in autophagy induction, because the Atg12 N terminus binds Atg17, a scaffolding subunit of the Atg1 autophagy induction complex [31].

## 3. Unexplored Ubiquitin-Like Folds in Atg5

**Figure 2:**
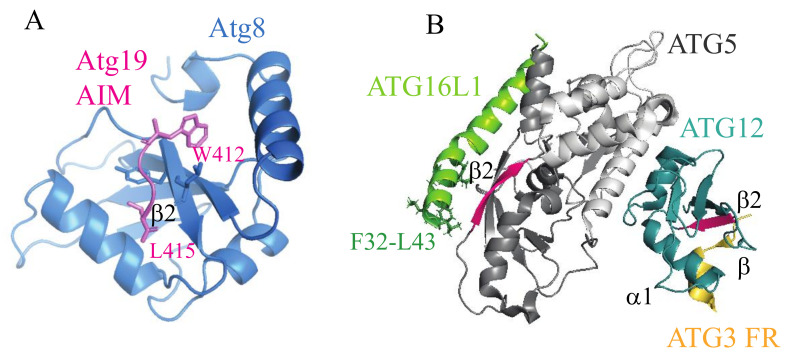
Binding of the autophagy related 8 (Atg8)-interacting motif (AIM)/microtubule associated protein 1 light chain 3 (LC3)-interacting region (LIR) sequences to the ubiquitin-like folds in the autophagy machinery. (**A**) The Atg8 ubiquitin-like (UBL) folds in a complex with the Atg19 AIM peptide (PDB ID: 2ZPN). Hydrophobic amino acid residues in the AIM motif (WEEL) are inserted into the two hydrophobic cavities (W and L site) on the flanking surface areas of the β2 strand. The Atg19 AIM tetrapeptide and the Atg8 β2 strand form an intermolecular β-sheet, a secondary structure found in all canonical AIM/LIR-Atg8/LC3/GABA type A receptor-associated protein (GABARAP) interactions. (**B**) The ATG3-ATG12–ATG5-ATG16N complex (PDB ID: 4NAW) in ribbon representation. When ATG3 binds to ATG12, the β2 strand (pink) of ATG12 forms an intermolecular β sheet with the β strand (orange) of the AADM_157_ tetrapeptide in the disordered region of ATG3 (ATG3 FR). The UBL-A domain (dark gray) on ATG5 possesses the β2 strand (pink). It remains to be elucidated if the ATG5 β2 forms an intermolecular β sheet with the β strand of the LIR motifs in autophagy receptors. Note that the ATG16L1 helical region spanning amino acid residues F32-L43 forms an amphipathic helix that inserts into a lipid bilayer [28,29], instead of binding to ATG5 that is seen in the crystal structure in the absence of membranes. FR, flexible region.

Numerous crystal structures show that all LIR peptides, as well as the AIM sequences, follow the same binding principle when they interact with Atg8/LC3/GABARAP. The UBL fold of Atg8-family proteins creates two hydrophobic pockets (called W and L sites) on the flanking surface area of the β2 strand, and each AIM/LIR tetrapeptide, in an intrinsically disordered domain, adopts the β strand to form an intermolecular β-sheet with the β2 of Atg8 homologs (Figure 2A) [4,32]. This binding mechanism is not entirely unique in autophagy. A recent crystallographic study of the human E3-like enzyme in a complex with ATG3 revealed that ATG12 shares with LC3 not only the UBL fold, but also a mechanism for the binding of a rope-like structure of its binding partner [26]. Specifically, a tetrapeptide (AADM_157_) in an intrinsically disordered loop of ATG3 adopts a β strand and forms an intermolecular β sheet with the β2 strand of ATG12 (Figure 2B). The UBL fold of human ATG12 creates a hydrophobic cavity on the surface of the β2 strand (PDB ID: 4NAW) that corresponds to the L site in LC3 and that fits M157 of the ATG3 tetrapeptide. There is no cavity on ATG12 corresponding to the LC3 W site. Similarity of the hydrophobic pockets of LC3 and ATG12 was interpreted to indicate a common ancestor protein from which both UBL folds evolved [26].

It is often overlooked that Atg5/ATG5 is another autophagy structure that adopts, in part, a ubiquitin-like fold. The protein is composed of two UBL domains connected by a single helical-rich domain (PDB ID: 2DYM or 2DYO and 4NAW) [26,33]. As mentioned in the previous section, Atg5/ATG5 was recently found in a study by Fracchiolla et al. to bind the AIM/LIR motifs of autophagy receptors [30]. The similarity of LC3 and ATG12 in binding of disordered regions opens a question of whether the UBL domain(s) in Atg5/ATG5 may also create hydrophobic pockets on the flanking surface area of the β2 strand for binding of the LIR/AIM tetrapeptide via an intermolecular β sheet, in analogy to Atg8/LC3/GABARAP. Fracchiolla et al. proposed one AIM-binding site away from the β2 strand in Atg5 and the other AIM-binding area on the Atg5 surface that requires movement of the Atg12 molecule to expose the hydrophobic surface for AIM binding [30]. However, such a movement of Atg12 is unlikely because of an extended interface between Atg5 and Atg12 that is important for the E3-like activity [23,24,25], and that was suggested to maintain Atg12 in a fixed position [34]. Thus, it remains unclear what type of structure the AIM/LIR tetrapeptide adopts when it interacts with Atg5/ATG5.

The crystal structure of the human ATG3-ATG12–ATG5-ATG16N (PDB ID: 4NAW) complex (Figure 2B), as well as the Atg12–Atg5-Atg16N complex from yeast (PDB ID: 3W1S, not depicted in Figure 2) shows a long β2 strand in the UBL-A, but not UBL-B domain of Atg5/ATG5. Does the AIM/LIR motif of autophagy receptors form an intermolecular β-sheet with the β2 strand of Atg5/ATG5, as it does with the β2 strand of Atg8/LC3/GGABARAP? Apparently, this question remains unresolved, and represents a research potential for future crystallographic studies. Along these lines, there are other questions in regard to the UBL folds in Atg5/ATG5. For example, why does Atg5/ATG5 possess two UBL domains? Another question is whether the presence of the well-formed β2 strand in Atg5/ATG5 UBL-A predisposes this domain for binding to the AIM/LIR. If so, what is the purpose of UBL-B? Apparently, autophagy-specific UBL folds have functional potentials, which, once elucidated, can reveal unknown mechanisms in autophagy.

## 4. Complex Interplay between the Ubiquitin-Proteasome System and Autophagy

Eukaryotic cells maintain protein homoeostasis by utilizing two pathways for protein degradation, the UPS and autophagy. The UPS targets individual short-lived proteins, whereas autophagy clears cells of long-lived proteins, protein complexes and protein aggregates. The UPS and autophagy were initially considered as two independent degradative pathways. However, for more than a decade, various studies have reported discoveries demonstrating a significant crosstalk between the ubiquitin-proteasome system and autophagy [35,36,37,38,39,40,41,42]. An interplay between these two degradative pathways occurs at multiple levels. One mechanism of communication is that changes in one proteolytic pathway induce changes in the activity of the other pathway. One of the first proteins discovered to be associated with this mechanism is HDAC6 (histone deacetylase 6), a microtubule-associated enzyme mediating autophagy induction in response to impaired UPS function [43]. Cancer cells treated with proteasome inhibitors also upregulate autophagy [44] and knockdown of two proteasomal ubiquitin receptors, PSMD4/S5a and ADRM1, in HeLa cells promotes compensatory SQSTM1/p62-mediated autophagy that clears accumulated polyubiquitinated substrates [45].

Further mechanisms activating autophagy due to inhibition of the UPS in more complex eukaryotes are N-terminal arginylation of endoplasmic reticulum chaperones, TP53/p53 (tumor protein p53) accumulation in the nucleus [37] and the unfolded-protein response [46]. Specifically, arginylation ensures recognition of ER-residing chaperones by the ZZ domain of SQSTM1/p62, leading to its conformational change, oligomerization and targeting to autophagy. In the case of TP53, a subpopulation of this protein accumulating in the nucleus acts as a transcription factor for autophagy-related genes [37]. Evidence for an opposite shift, that is autophagy-to-proteasome, is rather weak [35,38]. Some studies from yeast and human cells suggest that the proteasome is activated in response to pharmacological or genetic disruption of autophagy [47,48]. However, this view is contradicted by the finding that inhibition of autophagy in HeLa cells causes impaired UPS function due to accumulation of SQSTM1/p62, which would normally be degraded by autophagic clearance. Ubiquitinated substrates are sequestered by accumulated SQSTM1/p62, causing delayed delivery to the proteasome with otherwise unaffected activity [49]. A suggestion that some autophagic substrates are too large to be degraded by the proteasome is another plausible argument against bi-directional compensation in this direction [38].

A second aspect of communication between the UPS and autophagy is when a component of one degradative pathway regulates a subunit of, or becomes a substrate for, the other pathway. For instance, Atg16 in the slime mold *D. discoideum* can directly bind 19S proteasomal subunits Psmd1 and Psmd2 for lysosomal degradation [50]. Conversely, the 20S core proteasomal cylinder regulates autophagosome-lysosome fusion by degrading two autophagic soluble NSF attachment protein receptor (SNARE) proteins in human cells, SNAP29 (synaptosome-associated protein 29) and STX17 (syntaxin 17), involved in the fusion process [51]. Another example of this type of regulation is seen with the involvement of a deubiquitinating enzyme, USP14 (ubiquitin-specific peptidase 14). This protein directly interacts with the autophagy regulator UVRAG (UV radiation resistance associated), prolongs its half-life and upregulates the autophagy flux [52]. In contrast, USP14 activity that removes activating ubiquitins from BECN1 (beclin 1) downregulates autophagy [53]. Thus, the USP14–UVRAG axis has an opposite effect to the USP14–BECN1 axis. Proteaphagy, autophagic degradation of dysfunctional proteasomes [54,55], is also an important example of cross-regulation (described in detail below).

Other means of crosstalk of the two degradative pathways are chaperones and co-chaperones. For instance, decreased expression of the co-chaperone BAG1 (BAG cochaperone 1) at the expense of the expression of the co-chaperone BAG3 was proposed to promote a switch from proteasomal to autophagic degradation [56]. Another example is Cdc48/p97, a ubiquitin-binding chaperone that is regulated by many co-factors and that is an essential component of the UPS [57]. This chaperone is also important for the function of the autophagy pathway in yeast and mammals, and is indispensable for several types of selective autophagy in yeast [58].

The UPS and autophagy also communicate via sharing cellular components. For example, the transcription factor FOXO3 (forkhead box O3) participates in transcriptional co-regulation of both proteasomal and autophagic degradation [59]. The E3 ubiquitinase PRKN (parkin RBR E3 ubiquitin protein ligase) marks substrates for the proteasome but plays also an important role in mitophagy [60]. A major point where components are shared, but then substrates are specifically directed to a particular pathway, is the ubiquitination machinery: modifying various substrates with different types of ubiquitin moieties can determine which degradative pathway is utilized [61].

One type of substrate that illustrates this ubiquitin-dependent divergence is misfolded proteins, which can be degraded by either autophagy or the UPS. What are the molecular mechanisms and determinants that decide which pathway will be used? The type of linkage between ubiquitin moieties in a chain, known as the ubiquitin code [61], used to be considered a single key factor. The lysine (K) 48 linkage was proposed to be utilized by the UPS, whereas K27- or K63-linked ubiquitin chains were thought to mark protein aggregates or mitochondria for degradation by autophagy [62,63]. In agreement with this hypothesis, the K63 linkage is preferentially recognized by SQSTM1/p62 or NBR1, receptors acting in autophagy on ubiquitinated substrates [64]. However, increasing evidence suggests that the ubiquitin code itself is much more complex [65], and may be involved only in pre-disposition to one or the other pathway. The ubiquitin code alone is insufficient as a single factor for the choice of degradative pathway. A later study of NBR1 showed that this receptor has no preference for K48-linked or K63-linked ubiquitin chains [66]. Furthermore, experiments using the baker’s yeast *S. cerevisiae* demonstrated that Dsk2, a UPS receptor for soluble ubiquitinated proteins, and Cue5, an autophagic receptor for insoluble protein aggregates, have no preference for K48-linked or K63-linked ubiquitin chains [67]. Instead, Dsk2 exhibits much higher affinity for ubiquitin than Cue5, because Cue5 needs to self-interact and assemble into higher-order oligomers to stably bind to ubiquitin. A similar phenomenon was observed for other autophagy receptors, such as SQSTM1/p62 and NBR1 [68].

Thus, ubiquitin-binding receptors are additional players in the navigation of ubiquitinated substrates to the UPS or autophagy. These receptors are regulated via post-translational modifications (PTMs) or by intra- and/or inter-molecular interactions of receptor domains. Both types of regulation affect oligomerizations status, and, thereby, affinity of receptors for ubiquitin. The most common PTMs are ubiquitination and phosphorylation. These modifications, summarized in a recent review [41], on ubiquitin-binding receptors positively or negatively regulate their function in the UPS and autophagy, and thereby, affect the choice of degradative pathway. Intra- and inter-molecular interactions of receptors involve their major functional domains. With the exception of Cue5-TOLLIP (CUET) proteins, which bind ubiquitin via a coupling of ubiquitin conjugation to ER degradation (CUE) domain, most ubiquitin-binding receptors acting in the UPS or autophagy possess a ubiquitin-associated domain (UBA), a tightly-packed fold of approximately 40 amino acids comprised of three α-helices separated by two flexible regions. The first and third helix make hydrophobic contacts with a ubiquitin molecule. Studies show that UBA domains of human or yeast ubiquitin-binding receptors are engaged in intra- or inter-molecular interactions with their UBL or PB1 domains, and thereby, modulate an oligomeric state [41]. For example, Dsk1 from *S. cerevisiae* dimerizes through the UBA–UBL domain interaction [69,70]. In principle, ubiquitin and UBL or PB1 domains competing for the interaction with UBA can open or lock a receptor in a conformation that affects an oligomerization state, which in turn influences its involvement in the proteasomal or autophagic pathway. The consequence of this mechanism for misfolded proteins is that single polypeptides are degraded by the proteasome, whereas misfolded protein aggregates require oligomerized receptors for autophagic clearance by the vacuole/lysosome.

**Figure 3:**
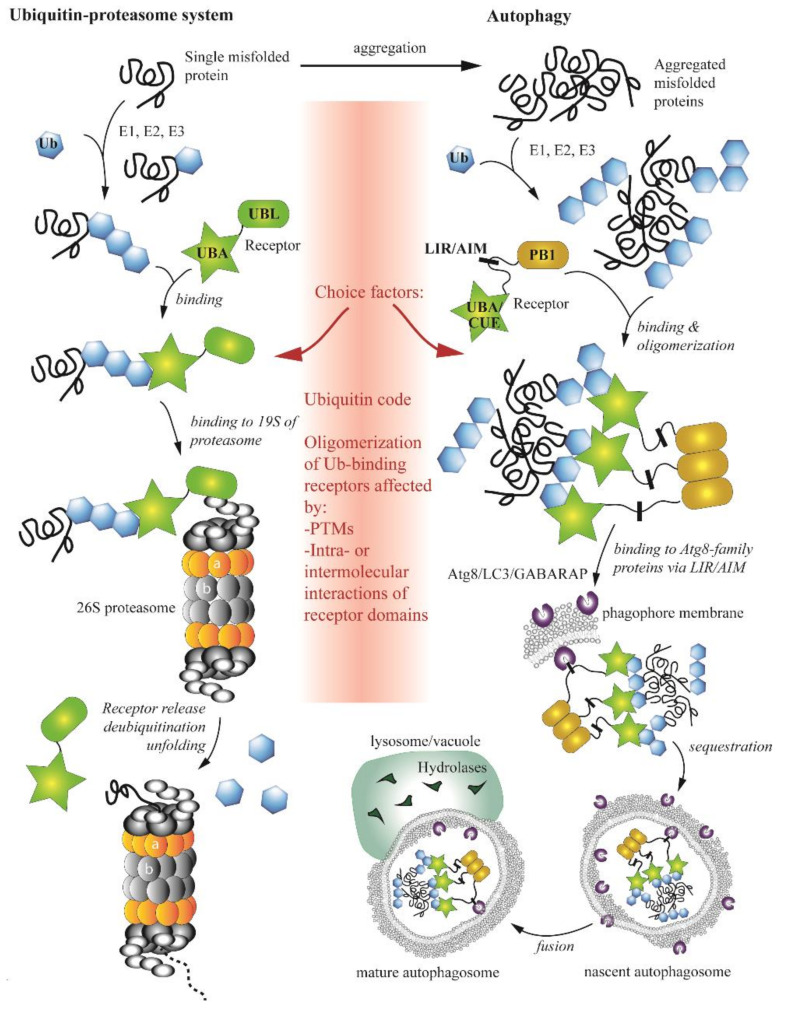
Degradation of misfolded proteins by the UPS and autophagy. Misfolded proteins are modified by the ubiquitination machinery involving the E1-E2-E3 enzymatic cascade. In the UPS (*left*), a mono- or poly-ubiquitin moiety on a single misfolded protein is recognized by a ubiquitin-associated (UBA) domain of a ubiquitin-binding receptor acting in the UPS, for example, Dsk2 in yeast. This complex is targeted to the 26S proteasome, where 19S subunits recognize a ubiquitin-like (UBL) domain of a receptor. After receptor release, deubiquitination, and unfolding of a misfolded protein, the 20S core cylinder composed of α and β subunits loads the protein for degradation. In autophagy (*right*), ubiquitin chains of misfolded protein aggregates are recognized by UBA domains of receptors acting in autophagy, for example, Cue5 in yeast. Oligomerization of these receptors via their UBA and Phox and Bem1 (PB1) domains is essential in high-affinity binding of receptors to ubiquitin. At the same time, the LIR/AIM motif of each receptor must be accessible for binding to Atg8-family proteins, and, thereby, targeting the receptor-substrate complex to the autophagy machinery. Specifically, the LIR/AIM motif binds to two hydrophobic pockets on the surface of Atg8/LC3/GABARAP that decorate the phagophore membrane. Expansion of the phagophore ultimately leads to sequestration of the cargo (that is, substrates with their corresponding receptors) by the nascent autophagosome that is decorated on the inner and outer membrane by Atg8-family proteins. After release of various proteins from the outer membrane, the mature autophagosome fuses with a degradative organelle, the vacuole in yeast and plants, and lysosomes in more complex eukaryotes, where hydrolases break down the cargo. The major factors (*red*) affecting which pathway will be used for misfolded-protein degradation are the ubiquitin code and ubiquitin-binding receptors. Binding affinity of each receptor to ubiquitin is determined by its oligomerization status that is affected by post-translational modifications (PTMs) and/or interactions between UBA, and UBL and PB1 domains of receptors.

In conclusion, there is no single, specific signal targeting substrates to the UPS or autophagy. The two pathways are significantly intertwined on multiple levels (Figure 3), making the network of communication very complex. Despite all recent discoveries, we are only at the beginning of disclosing the UPS-autophagy interplay.

## 5. Ubiquitin and Selective Autophagy in Yeast

As mentioned above, the ubiquitin code and ubiquitin-binding receptors are essential factors to determine which pathway will be used for degradation. Besides the UPS, there are multiple types of selective autophagy exploiting ubiquitin-binding receptors to target cargos for degradation. Depending on the specific cargo that is targeted and digested, selective autophagy can be distinguished as mitophagy, pexophagy, aggrephagy, ribophagy, proteaphagy, nucleophagy and the cytoplasm-to-vacuole targeting (Cvt) pathway, etc. (see below for detailed discussions) [71].

Of note, the molecular mechanisms of selective autophagy in yeast and mammals are conserved to some extent. Mounting evidence suggests that the ubiquitination of cargos is a critical mechanism for selective autophagy [72,73,74]. In ubiquitination-dependent selective autophagy, the ubiquitin on the cargo is usually exposed to the cytosol and is captured by ubiquitin-binding receptors, through a ubiquitin-binding domain. The receptors can interact with the ubiquitin-like protein Atg8, or the homologous LC3/GABARAP proteins, through an AIM or LIR, and thus recruit cargo to the phagophore membrane [74]. The main difference between yeast and mammals in this process is whether the action of a scaffold protein is required. In yeast, the interaction between specific cargo receptors for selective autophagy and Atg8 often needs the assistance of the scaffold protein Atg11, whereas no scaffold protein has been reported in more complex eukaryotes [75]. It is noteworthy that in addition to the critical interaction with receptors and Atg8 in selective autophagy, Atg11 can also associate with Atg9, Ypt1, Atg20, Atg1, the Atg17-Atg31-Atg29 complex and the Atg12–Atg5 conjugation system [76], demonstrating its multiple roles in mediating cargo selection, membrane trafficking, autophagosome biogenesis and phagophore expansion. To cover the current knowledge regarding various types of selective autophagy, we summarize the E3 ligase, scaffold and receptor proteins for each type of selective autophagy in yeast (Table 1) and mammals (Table 2).

In the following sections, we mainly focus on the roles of ubiquitin and receptors in selective autophagy in yeast.

### 5.1. Mitophagy

Mitophagy describes the specific autophagy process that engulfs and degrades damaged or superfluous mitochondria. Atg32 is the key receptor protein identified in yeast in the process of cargo recognition in mitophagy [77]. Atg32 is a mitochondrial outer membrane protein, and it connects mitochondria with the phagophore assembly site (PAS), the location of phagophore nucleation, through its association with Atg11 and Atg8 during mitophagy. With regard to mitophagy, the role of ubiquitination in cargo selectivity has been more clearly revealed in mammalian cells by the elucidation of the PINK1-PRKN/PARK2/parkin-mediated mitophagy mechanism [78]. Although no direct evidence has shown that Atg32 is a ubiquitin-binding receptor, a recent study reported that Atg32 is ubiquitinated at K282 [79], allowing a precise control of mitophagy.

In addition, a ubiquitin-dependent regulatory mechanism of mitophagy is also suggested by the suppression effect of the Ubp3-Bre5 de-ubiquitination complex on mitophagy [80]. Interestingly, although mitophagy is upregulated when genes encoding components of the Ubp3-Bre5 complex are deleted, other types of selective autophagy, including ribophagy and the Cvt pathway, are impaired in the absence of Ubp3 or Bre5, implying an intriguing role of Upb3-Bre5 in regulating distinct types of selective autophagy.

### 5.2. Cvt Pathway

The Cvt pathway is a yeast-specific biosynthetic pathway that utilizes autophagic machinery to deliver resident hydrolases, such as Ape1, Ams1 and Ape4, to the vacuole. Precursor Ape1 (prApe1) and Ams1 oligomerize after synthesis, and subsequently bind as large complexes to the receptor Atg19. prApe1 binding occurs in a propeptide-dependent manner [81]. In contrast, Ape4 does not self-assemble into a complex, but still interacts with Atg19 at a site different from prApe1 and Ams1 [82]. The Atg19 receptor interacts with the scaffold protein Atg11 through Hrr25-dependent phosphorylation, to recruit the Cvt complex (i.e., the cargo bound to Atg19) to the PAS. Similarly, the paralog of Atg19, Atg34, also acts as a receptor for Ams1, but not prApe1 or Ape4, under starvation conditions [83].

Interestingly, although no evidence has demonstrated that Atg19 and Atg34 are ubiquitin-binding receptors, Atg19 is ubiquitinated in vivo, and the attached ubiquitin is constitutively removed by the Ubp3-Bre5 complex [84].

### 5.3. Pexophagy

Pexophagy selectively degrades peroxisomes, organelles that are responsible for multiple biological functions, including lipid metabolism, purine catabolism, bile acid synthesis, etc. Atg30 and Atg36 are the receptors for pexophagy in the methylotrophic yeast *Komagataella phaffii/*Pichia pastoris** and S. cerevisiae, respectively. Atg30 recognizes and binds to the peroxisomal membrane proteins Pex3 and Pex14 in *K. phaffii/*P. pastoris,** and Atg36 binds to Pex3 in S. cerevisiae [85]. Thus, Pex3 and Pex14 are considered to function in part as docking factors to localize Atg30 and Atg36 on peroxisomes. Notably, however, instead of affecting the docking of Atg30 on peroxisomes, disrupting the Atg30-binding domain of Pex3 impairs the interaction between Atg30 and Atg11 [86], suggesting an additional regulatory role of Pex3 in pexophagy.

In mammalian cells, induction of pexophagy leads to the accumulation of ubiquitinated PEX5 at the peroxisomal membrane, following by binding of the ubiquitin-binding receptor NBR1 [87]; however, there is no equivalent mechanism in yeast [88,89], suggesting that an alternative signal instead of ubiquitination exists to target peroxisomes in this organism.

### 5.4. Aggrephagy

Aggrephagy selectively clears protein aggregates containing poly-glutamatine (poly-Q). Unlike pexophagy, there is evidence for ubiquitin-dependent aggrephagy in yeast, and Rsp5 is the E3 ligase that is responsible for the ubiquitination of protein aggregates in this process. The ubiquitin-binding receptor for aggrephagy in yeast is Cue5, which possesses a ubiquitin-binding CUE domain and an AIM domain [90]. Interestingly, Rsp5 can be immunoprecipitated with Cue5, and Cue5 itself is ubiquitin-regulated by Rsp5 as well.

As ubiquitinated protein aggregates are common substrates for both aggrephagy and the UPS, the precise mechanism by which cells orchestrate the pathways is under debate and investigation [41]. As described previously, many factors, including the position of Lys residues, the length of ubiquitin chains, the specificity of the receptors and receptor oligomerization, play highly complicated roles in the interplay between the UPS and aggrephagy.

### 5.5. Ribophagy

Unlike other forms of selective autophagy, where cargo ubiquitination typically marks substrates for removal, ubiquitination of the 60S ribosome protein Rpl25 by the ribosome-associated E3 ligase Rkr1/Ltn1 in yeast cells protects ribosomes from being targeted by selective autophagy. Instead, de-ubiquitination of Rpl25 on the same site by the Ubp3-Bre5 de-ubiquitinase triggers selective engulfment of the large ribosomal subunit by ribophagy during nutrient starvation conditions [91]. Further studies show that the chaperone-like protein Cdc48 and its ubiquitin-binding adaptor Doa1/Ufd3, both of which interact with Ubp3-Bre5, are also required for efficient ribophagy [92]. *PRO1*, the gene encoding gamma-glutamyl kinase, which genetically interacts with *UBP3*, is also involved in ribophagy [93]. However, how de-ubiquitinated Rpl25 is recognized by the phagophore, and whether there is another conventional ubiquitinated-cargo-receptor pair existing, remain unknown. Intriguingly, the Ubp3-Bre5 complex is only required for 60S ribosome but not 40S ribosome degradation, suggesting there is another independent ribophagy pathway and posing the question of how subunits of ribosomes are recycled separately.

In mammalian cells, NUFIP1 (nuclear FMR1-interacting protein 1) was identified as a selective ribophagy receptor [94]. In conditions of MTOR (mechanistic target of rapamycin kinase) complex 1 (MTORC1) inhibition, NUFIP1 binds to and co-migrates with the 60S ribosomal subunit; however, the ligand, and the nature of any MTORC1-mediated modification of the ligand, remain unknown.

### 5.6. Proteaphagy

Proteaphagy is the autophagic degradation of proteasomes, which represents another major point of crosstalk between autophagy and the UPS. Proteaphagy in yeast has been observed under basal conditions, nitrogen starvation and chemically or genetically induced proteasome inactivation. Proteaphagy during both nitrogen starvation and proteasome inhibition depends on Snx4/Atg24, which cooperates with sorting nexins Snx41 and Snx42 to mediate the nuclear export and agglomeration of proteasomes into cytoplasmic puncta before targeting to the vacuole [95]; however, the mechanisms in these two conditions are quite different. Following proteasome inactivation, proteasomes undergo extensive ubiquitination by an unknown E3 ligase, and accumulate in cytoplasmic insoluble protein deposits (IPODs) through chaperone Hsp42 delivery [55]. The receptor Cue5 binds to ubiquitinated proteasomes via its CUE domain, and to Atg8 via its AIM domain, thereby tethering aggregated proteasomes to the expanding phagophore [96]. In contrast, nitrogen deprivation-induced proteaphagy depends on the core autophagy machinery but not on Cue5 or Hsp42. In addition, the 20S proteolytic core particles (CPs) and 19S regulatory particles (RPs) of the proteasome are dissociated and separately targeted in nitrogen starvation-induced proteaphagy. The de-ubiquitinating enzyme complex Ubp3-Bre5 promotes autophagy of CPs but not RPs, and whether other receptors are involved is as yet unknown [97]. Intriguingly, during carbon starvation, the proteasome holo-complexes dissociate to CPs and RPs, which subsequently are exported to the cytoplasm with the aid of Blm10 and Spg5 respectively, and sequestered in proteasome storage granules to protect them from autophagy [98].

In contrast to yeast, amino acid starvation-induced polyubiquitination of proteasomes in more complex eukaryotes mostly involves RP subunits RPN1, RPN2, PSMD4/RPN10 and ADRM1/RPN13 [99]. This ubiquitination is essential for sequestration by the phagophore through the receptor SQSTM1/p62 via its ubiquitin-associated domain.

## 6. Ubiquitination in the Regulation of Autophagy

**Figure 4:**
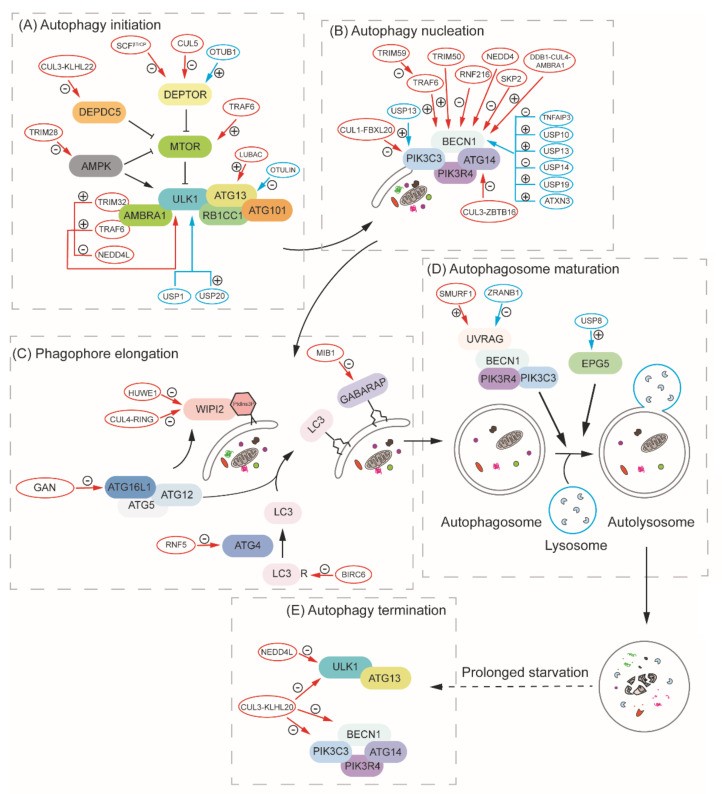
Ubiquitination in regulating autophagy-related proteins. All of the processes and proteins illustrated are shown for mammalian cells. (**A**) Autophagy initiation: autophagy is initiated by the inhibition of MTOR (mechanistic target of rapamycin kinase). Another energy-sensitive kinase, AMP-activated protein kinase (AMPK), is also involved in autophagy initiation through inhibiting MTOR and phosphorylating ULK1 (unc-51 like autophagy activating kinase 1) followed by the formation of a ULK1-ATG13-RB1CC1 (RB1 inducible coiled-coil 1)-ATG101 complex. During this process, MTOR, MTOR regulators, including DEPTOR (DEP domain containing MTOR interacting protein) and DEPDC5 (DEP domain containing 5, GATOR1 subcomplex subunit), AMPK, ULK1 and ATG13 can be regulated by ubiquitination and de-ubiquitination, which will further affect autophagy initiation. (**B**) Autophagy nucleation: following autophagy initiation, PtdIns3P, which is critical for the localization of phosphatidylinositol-3-phosphate (PtdIns3P)-binding proteins and the further recruitment of other ATG proteins, is generated by a phosphatidylinositol 3-kinase (PtdIns3K) complex at the phagophore. Some of the components of this PtdIns3K complex, including PI3KC3/VPS34 (phosphatidylinositol 3-kinase catalytic subunit type 3), BECN1 (beclin 1) and ATG14 can be ubiquitinated, and the modification on these proteins will affect their stability or function and further regulate autophagy activity. (**C**) Phagophore expansion: WIPI2 (WD repeat domain, phosphoinositide interacting 2), a PtdIns3P-binding protein, is recruited to the phagophore, which is followed by LC3 lipidation. The latter requires the function of two ubiquitin-like conjugation systems, which have been discussed in detail in the previous sections. For the purpose of better indicating how the ubiquitination system regulates phagophore expansion, we only show part of the process, including LC3 C-terminal processing by ATG4 and its conjugation to PE by the ATG12–ATG5-ATG16L1 complex. Ubiquitination on WIPI2, ATG16L1, ATG4, LC3 and GABARAP promotes their degradation and negatively regulates autophagy. (**D**) Autophagosome maturation: the phagophore expands and matures into a double-membrane structure termed an autophagosome, which will ultimately fuse with a lysosome. This process is reported to be facilitated by a UVRAG (UV radiation resistance associated)-containing complex and EPG5. These proteins can be regulated by ubiquitination and de-ubiquitination, thereby regulating autophagosome maturation. (**E**) Autophagy termination: after prolonged starvation, autophagy is terminated by the downregulation of the ULK1 and PtdIns3K complexes, which is mediated by the ubiquitination and degradation of some of the components in these two complexes, including ULK1, BECN1 and PI3KC3/VPS34. E3 ubiquitin ligases are shown in red circles and deubiquitinating enzymes are in blue. “+” indicates that the ubiquitination or de-ubiquitination enhances the stability or promotes the activity of the target protein; “-” refers to the degradation or functional inhibition of the substrate. AMBRA1, autophagy and beclin 1 regulator 1; ATXN3, ataxin 3; CUL3, cullin 3; DDB1, damage specific DNA binding protein 1; EPG5, ectopic P-granules autophagy protein 5 homolog; FBXL20, F-box and leucine rich repeat protein 20; GAN, gigaxonin; HUWE1, HECT, UBA and WWE domain containing E3 ubiquitin protein ligase 1; KLHL22, kelch like family member 22; LUBAC, linear ubiquitin chain assembly complex; MIB1, mindbomb E3 ubiquitin protein ligase 1; NEDD4L, NEDD4 like E3 ubiquitin protein ligase; OTUB1, OUT deubiquitinase, ubiquitin aldehyde binding 1; PIK3R4, phosphoinositide-3-kinase regulatory subunit 4; RNF216, ring finger protein 216; SKP2, S-phase kinase associated protein 2; SMURF1, SMAD specific E3 ubiquitin protein ligase 1; TNFAIP3, TNF alpha induced protein 3; TRAF6, TNF receptor associated factor 6; TRIM32, tripartite motif containing 32; USP1, ubiquitin specific peptidase 1; ZBTB16, zinc finger and BTB domain containing 16; ZRANB1, zinc finger RANBP2-type containing 1.

Protein degradation by autophagy and the UPS is crucial for proteostasis in the cell. Because the proteins carrying out these two pathways also need to be maintained/induced to a proper level in response to different conditions, it is not surprising that components or regulators of one system are degraded/recycled by the other. We have discussed the example of proteaphagy, in which entire proteasomes are the substrates of selective autophagy. Similarly, Atg proteins and their regulators can be ubiquitinated and subjected to proteasomal degradation by the UPS. Below, we review how ubiquitination serves as a means to control autophagic activity. Because there are only a few studies done in yeast, we will mainly focus on mammalian cells in this section (Figure 4).

### 6.1. The Ubiquitination System and Autophagy Initiation

In yeast, the target of rapamycin complex 1 (TORC1), which includes either Tor1 or Tor2, Kog1, Lst8 and Tco89, works as the master regulator of cell growth and metabolism [100]. Under nutrient-rich conditions, TORC1 inhibits autophagy through phosphorylating Atg13, which regulates its interaction with Atg1. The inhibition of TORC1 activity, for instance, by starvation or rapamycin, leads to the dephosphorylation of Atg13 and the assembly of the Atg1-Atg13-Atg17 complex, which initiates autophagy [101]. The corresponding mammalian rapamycin-sensitive MTORC1 also works as the master regulator of autophagy. MTORC1 consists of three major parts: MTOR, RPTOR/RAPTOR (regulatory-associated protein of MTOR complex 1) and MLST8 (MTOR-associated protein, LST8 homolog) [102,103,104]. The activity of MTORC1 can be regulated by the nutrient status of the cell. Nutrient sufficiency activates lysosome-localized RRAG (Ras related GTP binding) GTPases, which will bind to RPTOR and recruit the complex to the lysosome for its activation by RHEB (RAS homolog, mTORC1 binding) [105]. Activated MTORC1 phosphorylates both ATG13 and ULK1/2 (homolog of yeast Atg1), inhibiting the kinase activity. In response to starvation, MTORC1 is inactivated, leading to dephosphorylation of ULK1. At this point, ULK1 will phosphorylate ATG13, RB1CC1/FIP200 and ATG101, and initiate autophagy [106,107,108,109,110].

AMP-activated protein kinase (AMPK), another important energy-sensing kinase, is activated by metabolic stress or ATP consumption and promotes the catabolic pathway. In accordance with this, the yeast AMPK homolog, Snf1, is a positive regulator of nitrogen-starvation-induced autophagy, possibly through its phosphorylation of Atg1, and is essential for glucose-starvation-induced autophagy [111,112]. In mammalian cells, AMPK also works as an autophagy regulator through two mechanisms. When the energy level is low, AMPK is activated and phosphorylates TSC2 (TSC complex subunit 2) and promotes its activity as a GTPase-activating protein, which will inactivate RHEB, thereby inhibiting MTOR activation [113]. Meanwhile, AMPK phosphorylates RPTOR, which is another way to inhibit MTORC1 activity [114]. Besides the inhibition of MTORC1, AMPK also directly phosphorylates ULK1, which induces the formation of the ULK1 complex and autophagy initiation [115]. Overall, the initiation of autophagy requires the formation of the ULK1 complex, which is regulated by the energy-sensing kinases, MTORC1 and AMPK. Both of these kinases, as well as some of the components of the ULK1 complex, can be regulated by ubiquitination, and we will discuss how ubiquitination regulates autophagy initiation in the following subsections.

#### 6.1.1. Ubiquitination, Tor and MTOR Complex 1

Using a temperature-sensitive mutant yeast, Tor2, which is deficient in binding to Kog1, resulting in its degradation and the inactivation of TORC1, Hu et al. found that overexpression of ubiquitin prevents the degradation of Kog1, attenuates TORC1 inactivation and suppresses the growth defect seen at the non-permissive temperature [116,117]. Interestingly, the regulation of TORC1 activity by ubiquitin is not mediated by the normal polyubiquitination, but possibly through the non-covalent binding between ubiquitin and Kog1. However, how ubiquitin protects Kog1 from degradation still remains unknown and needs further investigation [116,117].

In mammalian cells, MTORC1 can also be regulated by ubiquitination, but compared with yeast, the mechanism is more complex. As mentioned above, MTOR activation requires its recruitment to the lysosome by RRAG GTPases [105]. In response to amino acids, the E3 ubiquitin ligase TRAF6 (TNF receptor-associated factor 6) is recruited to MTORC1 by SQSTM1, and this interaction is necessary for the translocation of MTORC1 to the lysosome and its subsequent activation. TRAF6 catalyzes K63-linked polyubiquitination of MTOR and regulates MTORC1 activation. Along these lines, the deletion of *TRAF6* leads to enhanced autophagic flux [118]. Recently, it was reported that the CUL3 (cullin 3)-KLHL22 (kelch-like family member 22) E3 ligase promotes K48-linked polyubiquitination and degradation of DEPDC5 (DEP domain-containing 5, GATOR1 subcomplex subunit), which inhibits RRAG GTPase activity under nutrient-deprivation conditions. Therefore, KLHL22 is critical for the activation of MTOR and works as a negative regulator of autophagy [119].

In addition to the major components of MTORC1 mentioned above, DEPTOR (DEP domain containing MTOR-interacting protein) is also an important protein in the MTOR complex, and is able to inhibit MTOR kinase activity [120]. Therefore, MTOR and autophagy activity can also be regulated by the ubiquitination of DEPTOR. DEPTOR interacts with BTRC/βTrCP (beta-transducin repeat containing E3 ubiquitin protein ligase), an F-box protein responsible for the specificity of the Skp1, Cullin, F-box (SCF)-containing complex E3 ligase. In response to a growth signal, DEPTOR is phosphorylated by different kinases, including RPS6KB1/S6K1, RPS6KA1/RSK1 and CSNK1A1/CK1, resulting in ubiquitination by the SCF E3 ligase and degradation. The accumulation of DEPTOR upon BTRC knockdown promotes autophagy through inhibiting MTOR activity [121,122,123]. Additionally, DEPTOR is ubiquitinated by CUL5 and degraded under normal growth conditions, which will lead to the activation of MTOR and inhibition of autophagy. However, when autophagy is induced, AMBRA1 (autophagy and beclin 1 regulator 1) stabilizes DEPTOR by inhibiting CUL5 activity so that the inhibition of MTOR activity is reinforced [124,125]. Furthermore, OTUB1 (OTU deubiquitinase, ubiquitin aldehyde binding 1) is reported to interact with, remove the polyubiquitin chain from, and stabilize DEPTOR, which suppresses MTOR activity and ultimately regulates autophagy [126].

#### 6.1.2. Ubiquitination of ULK1

ULK1 ubiquitination is mediated by various E3 ubiquitin ligases. For example, ULK1 is regulated by the TRAF6 E3 ligase. Upon autophagy induction, MTOR inactivation leads to the dephosphorylation of AMBRA1 and its interaction with TRAF6, which catalyzes K63-linked ubiquitination and promotes ULK1 stabilization, self-association and function [127]. AMBRA1 also interacts with another E3 ligase, TRIM32 (tripartite motif-containing 32), which in turn binds ULK1 and promotes K63-linked polyubiquitination of ULK1 in an AMBRA1-dependent manner. TRIM32 is necessary for the activation of ULK1 and autophagy induction during atrophic stimuli, such as dexamethasone treatment [128]. Apart from the ubiquitin ligase itself, C1QBP/p32 (complement C1q binding protein), a chaperone-like protein, impairs K48-linked, but promotes K63-linked, polyubiquitination of ULK1, and is thereby important for ULK1 stabilization and the initiation of both autophagy and mitophagy [129].

Some types of ubiquitination result in ULK1 degradation. NEDD4L (NEDD4-like E3 ubiquitin protein ligase) ubiquitinates ULK1, promoting its degradation, whereas the deletion of *NEDD4L* stimulates autophagy activity [130]. A mitochondrially localized E3 ligase, MUL1 (mitochondrial E3 ubiquitin protein ligase 1), promotes selenite-induced mitophagy but, paradoxically, MUL1 can also lead to the polyubiquitination and degradation of ULK1 [48]. In *Caenorhabditis elegans*, UNC-51/ULK1 is ubiquitinated by RPM-1, an atypical RING E3 ubiquitin ligase, which drives the degradation of UNC-51 and restriction of autophagy in the nervous system [131]. Besides the E3 ligases mentioned above, ULK1 is also ubiquitinated by other enzymes, but this modification usually occurs during prolonged starvation and leads to the termination of autophagy (discussed in detail below).

De-ubiquitination also plays an important role in regulating ULK1 activity. USP20 can bind, de-ubiquitinate and stabilize ULK1 at a basal level and plays an important role in autophagy initiation. However, USP20 will release from ULK1 at a later stage after autophagy initiation, and this dissociation leads to enhanced ULK1 degradation [132]. ULK1 de-ubiquitination is also controlled by USP1, a de-ubiquitinating enzyme that removes the K63-linked ubiquitin chain. The deletion of *USP1* results in the formation of ULK1 insoluble aggregates, and impaired canonical autophagic flux [133].

As mentioned above, AMPK is also a key factor to maintain energy homeostasis by upregulating autophagy, and it can also be regulated by ubiquitination. In yeast, the SAGA (Spt-Ada-Gcn5-acetyltransferase) complex, a histone modifier, can de-ubiquitinate Snf1 in a Ubp8-dependent manner. The deletion of *UBP8* leads to the hyperubiquitination of Snf1, which corresponds to a decrease in Snf1 phosphorylation, and results in the degradation of Snf1 [134]. However, the physiological role of this regulation, for instance, whether the regulation of Snf1 through ubiquitination affects autophagy activity, is unclear. Similarly, in mammalian cells, AMPK can be regulated by the ubiquitination system, and this modification can further regulate autophagy activity. MAGEA3 (MAGE family member A3)/MAGEA6 are frequently reactivated in cancer cells and bind to the TRIM28 E3 ligase, which ubiquitinates PRKAA1/AMPKα1 (protein kinase AMP-activated catalytic subunit alpha 1), promoting the degradation of AMPK and the inhibition of autophagy [135]. One of the components of the ULK1 complex, ATG13, can also be regulated by ubiquitination. Recently, Chu et al. [136] reported that the linear ubiquitin chain assembly complex (LUBAC) and the de-ubiquitinating enzyme OTULIN (OTU deubiquitinase with linear linkage specificity) promote and remove the linear ubiquitin on ATG13, respectively. The linear ubiquitin on ATG13 is important for protecting it from being degraded, and therefore plays a role in autophagy initiation. Accordingly, in OTULIN knockdown cells, autophagy initiation is promoted; however, the prolonged existence of ubiquitinated ATG13 inhibits autophagosome maturation. These findings together demonstrate that the ubiquitination system plays a critical role in regulating autophagy initiation.

### 6.2. The Ubiquitination System and Autophagy Nucleation

Autophagy nucleation refers to the mobilization of groups of molecules to the phagophore assembly site, which will further expand to form the phagophore and sequester autophagic cargos. In yeast, the class III phosphatidylinositol 3-kinase (PtdIns3K) complex is one of the key factors that are recruited to the PAS upon initiation. This complex includes five distinct proteins: the lipid kinase Vps34, the regulatory kinase Vps15, Vps30/Atg6, Atg14 and Atg38, which together generate phosphatidylinositol-3-phosphate (PtdIns3P) to direct the localization of some PtdIns3P-binding proteins and the further recruitment of other autophagy-related proteins [137]. In mammalian cells, the mechanism is similar: autophagy nucleation also depends on a PIK3C3/VPS34 (phosphatidylinositol 3-kinase catalytic subunit type 3)-containing PtdIns3K complex, including PIK3R4/VPS15 (phosphoinositide-3-kinase regulatory subunit 4), BECN1, ATG14 and NRBF2 [138]. Several studies show that some of these proteins can be regulated by post-translational modification, including ubiquitination, which further controls autophagy activity [139,140,141,142,143,144,145].

#### 6.2.1. Ubiquitination of PIK3C3/VPS34

The CUL1-SKP1 (S-phase kinase-associated protein 1) complex, with its adaptor FBXL20 (F-box and leucine-rich repeat protein 20), which interacts with PIK3C3/VPS34, mediates the ubiquitination and degradation of PIK3C3/VPS34 during DNA-damage-induced mitotic arrest, thus suppressing autophagy [146]. Moreover, Liu et al. reported that in *C. elegans*, CHN-1, an E3 ubiquitin ligase, interacts with VPS-34 and together with UBC-13 (an E2 ubiquitin-conjugating enzyme) and UEV-1 (a non-catalytic E2 variant), catalyzes the K63-linked ubiquitination of PIK3C3/VPS34 at K348 and K352, which is important for the stabilization of the protein: the loss of UBC-13 or CHN-1 partially impairs autophagy [147]. De-ubiquitination also plays an important role in regulating the activity of PIK3C3/VPS34. Recently, it was demonstrated that NEDD4/NEDD4-1 (NEDD4 E3 ubiquitin protein ligase) undergoes K29-linked auto-ubiquitination at K1279, recruiting USP13 to PIK3C3/VPS34, which reduces K48-linked ubiquitination of PIK3C3/VPS34 at K419 and promotes its stabilization. Therefore, NEDD4 functions as a positive regulator of autophagy by targeting PIK3C3/VPS34 [148].

#### 6.2.2. Ubiquitination of BECN1

TRAF6 can catalyze K63-linked ubiquitination of BECN1 at K117, which inhibits its interaction with BCL2 and facilitates lipopolysaccharide-induced autophagy [149]. Furthermore, the K63-linked ubiquitination mediated by TRAF6 can be inhibited by TRIM59 in non-small cell lung cancer cells. TRIM59 promotes the K48-linked ubiquitination and degradation of TRAF6, thereby inhibiting the ubiquitination of BECN1 and complex formation with PIK3C3/VPS34 [150]. Another protein from the TRIM family, TRIM50, also catalyzes the ubiquitination of BECN1 in a K63-dependent manner and induces its interaction with ULK1, thus functioning as a positive regulator of autophagy [151].

AMBRA1 interacts with BECN1 to regulate autophagy [152]. DDB1 (damage specific DNA binding protein 1)-CUL4 E3 ubiquitin ligase complex, together with the receptor AMBRA1, contributes to the K63-liked ubiquitination of BECN1. The K63-linked ubiquitination on K437 of BECN1 enhances its interaction with PIK3C3/VPS34 to promote the latter’s lipid kinase activity [153]. Moreover, RNF216 (ring finger protein 216), an E3 ubiquitin ligase, interacts with BECN1 and drives its K48-linked ubiquitination, which promotes the degradation of BECN1 and inhibits autophagy [154]. Additionally, NEDD4 also mediates the ubiquitination of BECN1, but the role of ubiquitination mediated by NEDD4 is controversial. One study showed that NEDD4 controls K11- and K63-linked polyubiquitination, with the former enhancing the degradation of BECN1 [155]. However, another study demonstrated that NEDD4 interacts with BECN1 and mediates its K6- and K27-linked ubiquitination, which stabilizes BECN1 and promotes autophagy. Meanwhile, the knockdown of NEDD4 results in enhanced K48-linked ubiquitination of BECN1 and impaired autophagy [156]. Therefore, the role of NEDD4-mediated ubiquitination of BECN1, whether it depends on different conditions, and how it affects autophagy, still need further investigation. Recently, another E3 ligase, SKP2, was found to execute K48-linked polyubiquitination at K402 of BECN1, which induces its degradation and inhibits autophagy [157].

De-ubiquitinating enzymes are also involved in regulating BECN1 activity. Contrary to TRAF6, the de-ubiquitinating enzyme TNFAIP3/A20 (TNF alpha induced protein 3) reduces K63-linked ubiquitination of BECN1, inhibiting the autophagy induced by lipopolysaccharide [149]. Ubiquitin on BECN1 can also be removed by USP10 and USP13, which maintains the stability of the BECN1- PIK3C3/VPS34 complex, and BECN1 in turn can regulate the stability of USP10 and USP13 [158]. Jin et al. reported that USP19 stabilizes BECN1 by removing the K11-linked ubiquitin chains of BECN1 at K437, thus positively regulating autophagy [159]. In contrast, USP14 is responsible for moving the K63-linked ubiquitination on BECN1, resulting in autophagy inhibition [160]. Furthermore, another de-ubiquitinating enzyme, ATXN3 (ataxin 3), interacts with BECN1 and removes the K48-linked polyubiquitin chain from K402, which protects BECN1 from proteasome degradation, and enables autophagy [161].

Besides PIK3C3/VPS34 and BECN1, some other proteins in this complex can be regulated by ubiquitination. ATG14 is ubiquitinated by the ZBTB16 (zinc finger and BTB domain containing 16)-CUL3-RBX1/Roc1 (ring-box 1) E3 ubiquitin ligase complex in response to G-protein-coupled signaling, which leads to the degradation of ATG14 and the inhibition of autophagy [162]. In addition, AMBRA1 can be ubiquitinated in a K48-linked manner by RNF2 at K45, leading to the degradation of AMBRA1 and reduced PIK3C3/VPS34 activity [163]. Apart from the core components of the PtdIns3K complex, some of its regulators are also found to be regulated by ubiquitination. PRKN mono-ubiquitinates and stabilizes BCL2, which results in more interaction between BECN1 and BCL2, thus suppressing autophagy [164]. Moreover, it has been reported that the RAS-like GTPase RALB (RAS like proto-oncogene B) and its effector EXOC8/Exo84 (exocyst complex component 8) drive the assembly of the BECN1-PIK3C3/VPS34 complex [165], and USP33 can de-ubiquitinate RALB under starvation conditions to induce the formation of the RALB-EXOC8-BECN1 complex, which is crucial for autophagosome formation [166]. Therefore, the ubiquitination system provides diverse ways to regulate autophagy by stabilizing or inducing degradation of the PIK3C3/VPS34-BECN1 complex and its regulators.

### 6.3. The Ubiquitination System and Phagophore Expansion

After nucleation, one of the essential processes in autophagosome formation is the recruitment of the PtdIns3P-binding protein WIPI2 (WD repeat domain, phosphoinositide-interacting 2; a homolog of yeast Atg18), followed by ATG12–ATG5-ATG16L1 complex-mediated LC3 lipidation and phagophore expansion. In this section, we mainly focus on how these proteins that are involved in phagophore expansion are regulated by ubiquitination.

WIPI2 can be ubiquitinated by HUWE1 (HECT, UBA and WWE domain containing E3 ubiquitin protein ligase), which leads to the degradation of WIPI2 and autophagy inhibition. The HUWE1-mediated ubiquitination depends on WIPI2 phosphorylation by MTOR at S395, and this provides another explanation for how MTOR inhibits autophagy [167]. A more recent study demonstrated that WIPI2 is a substrate of CUL4-RING ubiquitin ligase, which is activated upon mitosis induction and promotes the K48-linked polyubiquitination of WIPI2 and its degradation, again inhibiting autophagy [168].

In the ubiquitin-like conjugation system, the protease Atg4 is necessary for priming Atg8 for lipidation by cleaving the last arginine residue to expose the penultimate glycine. In mammalian cells, there are four ATG4 proteins: RNF5 ubiquitinates ATG4B and induces its proteasome-mediated degradation, which further limits LC3 lipidation and basal autophagy activity [169]. ATG16L1, which interacts with ATG12–ATG5, is also essential for phagophore expansion [24], and triggers the binding of the complex to the membrane, specifying the location of LC3 lipidation [170]. Ubiquitination of ATG16L1 is mediated by the GAN (gigaxonin) E3 ligase, and the ubiquitination results in the degradation of ATG16L1. The deletion of *GAN* leads to the aggregation of ATG16L1, which impairs LC3 lipidation and autophagosome biogenesis [171]. LC3B can be monoubiquitinated and targeted for degradation by the ubiquitin-activating enzyme UBA6 (ubiquitin-like modifier-activating enzyme 6) and the hybrid ubiquitin-conjugating enzyme/ubiquitin ligase BIRC6 (baculoviral IAP repeat containing 6), which negatively regulates autophagy through reducing the amount of LC3B [172]. One of the Atg8-family proteins in mammalian cells, GABARAP, can be ubiquitinated by MIB1 (mindbomb E3 ubiquitin protein ligase 1), but the centriolar satellite protein PCM1 (pericentriolar material 1) binds GABARAP and protects it from MIB1-mediated ubiquitination and degradation, promoting GABARAP-positive autophagosome formation [173].

### 6.4. The Ubiquitination System and Autophagosome Maturation

As noted above, the expanding phagophore ultimately matures into a double-membrane autophagosome, which then fuses with a vacuole in yeast or a lysosome in mammalian cells for the degradation of the cargo. UVRAG is important in regulating autophagosome maturation by interacting with BECN1, PIK3C3 and RUBCN (rubicon autophagy regulator) [174,175,176]. A recent study showed that an E3 ubiquitin ligase, SMURF1 (SMAD specific E3 ubiquitin protein ligase 1), induces K29- and K33-linked polyubiquitin of UVRAG at K517 and K559, which inhibits its association with the inhibitor RUBCN, and thereby promotes autophagosome maturation. In contrast, the de-ubiquitinating enzyme, ZRANB1 (zinc finger RANBP2-type-containing 1) antagonizes SMURF1-mediated ubiquitination and functions as a negative regulator of autophagy [177]. In addition, EPG5 (ectopic P-granules autophagy protein 5 homolog) functions as a RAB7 (RAB7, member of the RAS oncogene family) effector responsible for the specificity of autophagosome and lysosome or late endosome fusion [178]. The de-ubiquitinating enzyme USP8 removes non-classical K63-linked ubiquitin chains from EPG5 at K252, which leads to a strengthened interaction between EPG5 and LC3. The overexpression of USP8 results in enhanced autophagy flux in both normal and starvation conditions in embryonic stem cells [179].

### 6.5. The Ubiquitination System and Autophagy Termination

Even though autophagy is essential in keeping the homeostasis of cells under stress conditions such as starvation, the failure of autophagy termination will also lead to cellular damage or death [180]. In addition to the MTOR reactivation that occurs in response to the autophagy-dependent release of intracellular nutrients from the lysosome, ubiquitination-mediated degradation of key proteins in autophagy also plays an important role in terminating this process. AMBRA1 is ubiquitinated by DDB1-CUL4 under nutrient-rich conditions, and this ubiquitination ultimately lowers the amount of AMBRA1. Upon starvation, DDB1-CUL4 transiently dissociates from AMBRA1, which then stabilizes DEPTOR and promotes autophagy through inhibiting CUL5 activity, as mentioned above. However, during prolonged starvation, DDB1-CUL4 re-associates with AMBRA1, driving the ubiquitination and degradation of this protein and leading to autophagy termination [124,125]. Furthermore, CUL3, together with its adaptor KLHL20, ubiquitinates ULK1 and controls the degradation of ATG13, PIK3C3/VPS34, BECN1 and ATG14 in a direct or indirect mechanism under conditions of prolonged starvation, therefore regulating the amplitude and termination of autophagy [181]. The NEDD4L E3 ubiquitin ligase also participates in autophagy termination. During prolonged starvation, NEDD4L mediates the ubiquitination of ULK1 at K925 and K933, which leads to the degradation of ULK1 [182]. These facts indicate that many ubiquitin enzymes cooperate to regulate the protein levels of several essential genes in autophagy during prolonged stress conditions, which contributes to autophagy termination.

### 6.6. Ubiquitination of Autophagy Regulators

Apart from the master regulators such as MTOR and AMPK, some downstream regulators, including transcription factors, control the expression of essential autophagy genes and keep autophagy activity at a proper level. Several transcription factors are regulated by ubiquitination; therefore, autophagy can be regulated by ubiquitination of these transcription factors.

In yeast, Ume6, together with the corepressor Sin3 and the histone deacetylase Rpd3, works as a negative regulator of *ATG8* transcription, and the deletion of *UME6* increases the level of Atg8 and autophagy activity [183]. Ume6 is reported to be a substrate of the E3 ubiquitin ligase APC/C^Cdc20^ (anaphase promoting complex-cell division cycle 20), which is required for the degradation of this protein. Even though in this study, the authors only showed the role of this ubiquitination in meiosis, the fact that Ume6-Cdc20 association is regulated by PKA, another kinase involved in autophagy control [184], implies that the ubiquitination of Ume6 may also function in autophagy regulation [185].

In mammalian cells, TP53/p53 (tumor protein p53) is a transcription factor that plays a dual role in regulating autophagy [186]. Nuclear-localized TP53 stimulates the transcription of several genes, such as *ATG2*, *ATG4*, *ATG7* and *ATG10*. Conversely, cytosolic TP53 suppresses autophagy through inhibiting AMPK and activating MTOR [187]. However, the stimulation of autophagy, such as occurs through rapamycin treatment and starvation, can induce the degradation of TP53, which depends on the E3 ubiquitin ligase MDM2/HDM2 (MDM2 proto-oncogene), and the inhibition of TP53 degradation inhibits autophagy activation [188].

Apart from TP53, the stability and activity of some other transcription factors that regulate autophagy are also controlled by ubiquitination in mammalian cells [189]. For instance, FOXO family proteins control the transcription of some *ATG* genes, and some of the FOXO proteins can be regulated by ubiquitination. SKP2, COP1 (COP1 E3 ubiquitin ligase) and MDM2 mediate the polyubiquitination of FOXO1 (forkhead box O1) and thus promote its degradation [190,191,192]. A de-ubiquitinating enzyme, USP7, removes the mono-ubiquitination on FOXO1 and negatively regulates its transcriptional activity [193]. In addition, FOXO3 can be ubiquitinated by MDM2 and SKP2, both of which mediate its degradation via the proteasome [194]. E2F1 (E2F transcription factor 1) can induce autophagy by stimulating the transcription of several *ATG* genes, including *ATG1* and *ATG5*, and the knockdown of E2F1 will impair DNA-damage-induced autophagy [193]. Two de-ubiquitinating enzymes, PSMD14/POH1 (proteasome 26S subunit, non-ATPase 14) and UCHL5/UCH37 (ubiquitin C-terminal hydrolase L5), remove K63-linked poly-ubiquitin chains on E2F1, leading to the stabilization and enhanced activity of E2F1, respectively [195,196]. However, it was reported by a later study that BIRC2/cIAP1 E3-ligase promotes the K63-linked poly-ubiquitination at K161/164 on E2F1, which is associated with DNA-damage-induced stabilization of this protein [197]. Therefore, the role of DNA-damage-associated K63-linked ubiquitination on E2F1 and the subsequent outcome need further study. Even though there is no report showing whether or how these ubiquitination modifications directly regulate autophagy, we cannot exclude the possibility that the ubiquitination of these transcription factors also plays a role in controlling autophagy activity. The ubiquitination status of these transcription factors under different conditions, and whether and how they affect the transcription of genes involved in autophagy, still need further investigation.

## 7. Conclusions and Perspectives

Autophagy and the UPS are the essential degradation programs for cellular homeostasis in all eukaryotes. These two pathways communicate with, compensate and regulate each other in a coordinated manner to efficiently and effectively maintain the intracellular catabolism at a proper level. Interestingly, ubiquitination, which has been initially considered to be a specialized tag for the UPS, is involved in regulating multiple aspects of autophagy, from the nucleation of the phagophore, to autophagosome fusion with the lysosome. From the structural point of view, adopting the ubiquitin architecture into the ubiquitin-like folds of three proteins (namely Atg8, Atg12 and Atg5 in *S. cerevisiae* and their homologs in more complex eukaryotes) playing a central role in the core autophagy machinery, illustrates the significance of this architecture for the autophagy pathway. The UBL fold of Atg12/ATG12 provides a base for binding of Atg3/ATG3, an interaction that is indispensable for efficient lipidation of Atg8-family proteins. The UBL folds of Atg8/LC3/GABARAP and Atg5/ATG5 create binding platforms for a vast number of receptors and adaptors that connect the autophagy machinery to other components in yeast and mammalian cells. Furthermore, two different subsets of receptors utilize two different motifs, AIM/LIR or ubiquitin-interacting motif/UIM, that can bind simultaneously to two different docking sites (LDS or UDS) on the UBL fold of Atg8-family proteins [96]. Such a complexity is further amplified by an intrinsically disordered nature of the Atg3/ATG3 or receptor and adaptor sequences, which bind to the autophagic UBLs. These intrinsically disordered regions offer tremendous potential for PTMs and structural plasticity, and, together with the UBLs, yield remarkable versatility that is available to the autophagy pathway, and that is worth exploring through further research.

Although autophagy was initially thought to be non-selective, tremendous studies in recent years have identified and uncovered the mechanisms of multiple selective autophagy pathways [71]. Ubiquitination is an indispensable signal to initiate some types of selective autophagy. In fact, the specificity of many selective autophagy pathways is determined by ubiquitination and the binding of receptor proteins. One intriguing question is what determines which pathway recycles the ubiquitinated substrate. Some studies suggest that the ubiquitin chain type is the deciding factor, but other studies identified the same ubiquitin chain type in both pathways [37,40,198]. The receptor recognition and binding partly accounts for the choice of autophagy, however, this still does not explain why the binding to receptors occurs before diverting the substrate into the UPS. Another possibility is that further post-translational modifications of the ubiquitin chains is part of the signal and is involved in the process of recognition and preferential binding. Further studies are needed to understand what is fully encoded by ubiquitin signaling. It will also be interesting to understand how the ubiquitinated substrates are selected to represent the entire organelle; that is, does part of the specificity come from the ubiquitinated cargo?

Numerous studies in the past have shown the involvement and importance of the UPS in the regulation of many cellular processes [199], including autophagy. This can occur through either direct turnover of Atg proteins or modulating upstream signals that control autophagy. Because autophagy often works as an adaptive response to environmental stimuli such as starvation, it is of great importance to understand how the UPS is integrated to regulate autophagy in a spatio-temporal correct manner. In some of the studies mentioned above, autophagy regulation through ubiquitination or de-ubiquitination of critical proteins is related to certain types of pathophysiologies, such as cancer and infectious diseases, some of which are valid drug targets. For instance, it was found that in breast tumors of patients, there is an increasing level of KLHL22 and a corresponding decreasing level of DEPDC5. More interestingly, the deletion of *KLHL22* suppresses the growth rate of breast cancer cells, and *KLHL22* knockout also prevents tumor growth in mice. All these findings indicate that KLHL22 may work as an oncogene, and the small molecules inhibiting its activity may work as drugs to treat breast cancer [119]. Additionally, K29- and K33-linked ubiquitination on UVRAG, which induces autophagosome maturation, promotes the degradation of EGFR and inhibits the growth of hepatocellular carcinoma. This study reveals the role of an autophagy-related ubiquitination on UVRAG in tumor growth, indicating another therapeutic potential [177]. Besides cancer, autophagy-related ubiquitination is also involved in other diseases. For example, TRIM32 positively regulates autophagy through ubiquitinating ULK1, whereas disease-associated mutant forms of the protein are deficient in promoting autophagy. This matches the defective autophagy seen in myoblasts derived from limb-girdle muscular dystrophy type 2H/LGMD2H patients, implying that TRIM32-dependent autophagy may have an important correlation with this disease [128]. Another study also shows that SKP2i, an SKP2 inhibitor, inhibits the replication of MERS-CoV (Middle East respiratory syndrome coronavirus) through enhancing the BECN1 level and autophagy activity, and these findings suggest the potential of some SKP2 inhibitors to be used in the clinical treatment of viral infection [157]. Still another example is that an E3 ligase, TNFAIP3, and ATG16L1, which participates in autophagy, have been found to be associated with inflammatory bowel disease in a genome-wide association study [200,201]; a recent analysis shows that these two proteins interact and regulate the level of each other in a compensatory way to maintain the integrity of the intestinal epithelial barrier, and the loss of both proteins leads to chronic intestinal inflammation [202]. Therefore, whether and how these different types of regulation are implicated in human diseases, and whether there exists the potential to target them in therapeutic strategies, are worth further study.

Although tremendous progress has been made in understanding the involvement of ubiquitination in autophagy, there are still many more questions that need to be addressed. One common question, as mentioned above, is the specific recognition of the substrates, which is mostly determined by E3 ligases. Therefore, in the journey of completing our understanding of the degradation network, it is of great significance to identify the E3 ligases involved in selective autophagy and the associated regulatory steps.

## Figures and Tables

**Table 1:** Cargo, receptor and E3 ligase of selective autophagy in yeast.

Pathway	Cargo	Receptors	E3 Ligase
Cytoplasm-to-vacuole targeting (Cvt) pathway	prApe1, Ams1 and Ape4	Atg19 and Atg34	-
Mitophagy	Mitochondria	Atg32	-
Pexophagy	Peroxisomes (*S. cerevisiae*)	Atg36	-
	Peroxisomes (*K. phaffii/P. pastoris*)	Atg30	-
Aggrephagy	Protein aggregates	Cue5	Rsp5
Ribophagy	Ribosome	-	-
Proteaphagy	Proteasome	Cue5	-

**Table 2:** Cargo, receptor and E3 ligase of selective autophagy in mammals.

Pathway	Cargo	Receptors	E3 Ligase
Mitophagy	Mitochondria	SQSTM1/p62, BNIP3L/Nix, OPTN	PRKN/PARK2/ parkin
		FUNDC1, PHB2, CALCOCO2/NDP52	
Pexophagy	Peroxisomes	SQSTM1/p62, NBR1	PEX2
Aggrephagy	Protein aggregates	SQSTM1/p62, NBR1, TOLLIP	-
Ribophagy	Ribosome	NUFIP1	-
Proteaphagy	Proteasome	SQSTM1/p62	-

BNIP3L, BCL2 interacting protein 3 like; FUNDC1, FUN14 domain containing 1; NUFIP1, nuclear FMR1 interacting protein 1; PHB2, prohibitin 2; TOLLIP, toll interacting protein.

## Abbreviations

AIM	Atg8-interacting motif
Atg	autophagy-related
CPs	core particles
CUE	coupling of ubiquitin conjugation to ER degradation
Cvt	cytoplasm-to-vacuole targeting
LIR	LC3-interacting region
MTORC1	mechanistic target of rapamycin kinase complex 1
PAS	phagophore assembly site
prApe1	precursor aminopeptidase I
PtdIns3K	phosphatidylinositol 3-kinase
PtdIns3P	phosphatidylinositol-3-phosphate
PTMs	post-translational modifications
RPs	regulatory particles
UBA	ubiquitin-associated domain
UBD	ubiquitin-binding domain
UBL	ubiquitin-like
UPS	ubiquitin-proteasome system
